# Moral Disengagement Mechanisms in Image-Based Sexual Abuse Against Women: The Role of Age and Gender

**DOI:** 10.3390/bs16071047

**Published:** 2026-06-23

**Authors:** Jone Martínez-Bacaicoa, Román Ronzón-Tirado, Sophie McBain-Ritchie, Manuel Gámez-Guadix

**Affiliations:** 1 Biological and Health Psychology Department, Autonomous University of Madrid, 28049 Madrid, Spain; 2 Department of Psychology, Faculty of Health Sciences, University of Deusto, 48007 Bilbao, Spain

**Keywords:** non-consensual intimate image sharing, sextortion, image-based sexual abuse, technology-facilitated sexual violence

## Abstract

Image-based sexual abuse (IBSA) is an increasingly prevalent problem that disproportionately affects women. Understanding the psychological processes related to this behavior is essential for its prevention. Accordingly, the present study examines the activation of moral disengagement mechanisms in IBSA contexts by considering the role of gender and age across the lifespan. Specifically, by using a vignette-based methodology, this study investigates which moral disengagement mechanisms are activated in scenarios of sextortion and non-consensual intimate image sharing (NCIIS) involving male-perpetrated abuse against women. A sample of 2343 participants (68.2% women) aged 14–74 years (*M* = 25.86, SD = 9.96, *Mo* = 19) completed measures which assessed eight mechanisms of moral disengagement. The results indicated that men exhibited higher levels of moral disengagement than women in relation to both sextortion and NCIIS, with younger men reporting the highest levels. Gender differences were more pronounced for NCIIS (ηp^2^ = 0.085) than for sextortion (ηp^2^ = 0.043). With regard to age, older participants reported lower overall levels of moral disengagement in both scenarios, although age effects were comparatively small (ηp^2^ = 0.020–0.026). The minimization of consequences in sextortion was the only mechanism that remained relatively stable across ages. Analyses also revealed significant age × gender interactions, particularly for NCIIS (ηp^2^ = 0.016), indicating that moral disengagement among women remained at consistently lower levels, whereas initial gender differences between men and women decreased with age. These findings are consistent with prior literature which suggests that both sextortion and NCIIS constitute gendered forms of violence and highlight the importance of targeting young men in prevention and intervention efforts aimed at challenging the justifications underlying these behaviors.

## 1. Introduction

The development of digital technologies and social media platforms has transformed social dynamics and interpersonal relationships. While new opportunities for social connection are being created, novel forms of harm have also appeared (Mateos-Pérez et al., 2025; Santoniccolo et al., 2023). Among these, image-based sexual abuse (IBSA) has emerged as a serious and rapidly escalating problem (Martínez-Bacaicoa et al., 2024b; Umbach & Henry, 2025). This term describes various forms of behavior that involve the non-consensual creation, distribution or threatened distribution of intimate or sexual images without the individual’s consent (McGlynn et al., 2017). In the present study, we focus on two key forms of IBSA: non-consensual intimate image sharing (NCIIS), which involves distributing intimate or sexual images of a person without their consent (Said & McNealey, 2023), and sextortion, which occurs when the perpetrator threatens to distribute intimate images of a victim unless they comply with sexual, financial or reputational demands (Ray & Henry, 2025). This can apply to images that were originally taken or created consensually (e.g., within a relationship) or non-consensually (e.g., through hidden recording or artificial intelligence).

IBSA represents a significant and increasingly prevalent social problem. Recent cross-national evidence indicates that nearly 1 in 2 individuals have been exposed to non-consensual intimate images of another person (Umbach & Henry, 2025), and that more than 1 in 5 individuals have experienced some form of victimization (Umbach et al., 2025). Although research often reports similar victimization rates among men and women, there is increasing evidence that IBSA should be considered a gendered form of violence (Martínez-Román et al., 2026; Nygård et al., 2025; Pedersen et al., 2023; Powell et al., 2024). In this regard, research suggests that men are more likely to perpetrate all forms of IBSA (Ray & Henry, 2025; Umbach & Henry, 2025). Moreover, while individuals of any gender may be victimized, most sexual content distributed without consent depicts women’s bodies (Henry & Flynn, 2019). Gendered dynamics are also evident in sextortion, where male victims are more often subjected to financial demands, while female victims are more likely to face sexual demands (Wang, 2025). Together, these patterns help explain why the psychological and emotional consequences associated with IBSA victimization (Hellevik et al., 2025; Huber & Ward, 2025) tend to be particularly severe among women (Powell et al., 2024; Umbach et al., 2025). Therefore, IBSA should be understood not just as interpersonal harm but as embedded in gendered power relations that perpetuate violence against women. Importantly, adopting this perspective does not imply that gender is the only factor shaping experiences of IBSA, as other variables (e.g., age) may also influence how these experiences are understood and responded to.

The high prevalence of IBSA, together with the severity of its consequences, highlights the urgent need for prevention efforts. Advancing prevention requires a better understanding not only of the factors that may facilitate its perpetration, but also of the processes that may discourage intervention when these behaviors are witnessed (Kromann & Flynn, 2025; Mainwaring et al., 2026). This is especially relevant because the harm associated with IBSA often extends beyond the initial act and can be sustained or amplified by the responses of others. For example, the impact of NCIIS may vary depending on whether recipients decide to further distribute the image, remain passive, or take steps to interrupt the cycle of abuse (Harder, 2021; Schmidt et al., 2024). Similarly, when intimate images are used as a form of coercion or extortion, the reactions of those who are aware of the situation can also play a significant role in either increasing or reducing the impact of the abuse (Pacilli et al., 2025; Wang, 2025). This suggests that understanding and preventing IBSA requires attention not only to the abusive acts themselves, but also to the social and cognitive processes that may allow them to be tolerated, minimized, or left unchallenged.

In this context, moral disengagement may be a particularly useful construct for understanding the cognitive processes that allow IBSA to be justified (Bandura, 2016). Although prior research points to the relevance of moral disengagement in this domain (Martínez-Bacaicoa et al., 2024a; Pina et al., 2021), little is known about which specific mechanisms are more likely to be activated in IBSA scenarios. This study seeks to address this gap by examining how different mechanisms of moral disengagement are activated in IBSA situations, specifically NCIIS and sextortion, and whether these processes vary according to age and gender. Consistent with the gendered patterns described above, this study focuses on situations involving male-perpetrated IBSA against women, as this represents one of the most common victim–perpetrator configurations reported in the literature. The following sections outline the rationale for these aims.

### 1.1. Moral Disengagement

Moral disengagement refers to the psychological process by which a person suppresses their ethical standards in order to engage in harmful actions without feeling guilt or remorse (Bandura, 1978, 1990, 2016). This process operates through the selective activation of eight psychosocial mechanisms, which are organized into four clusters according to their locus within the behavioral episode (Bandura, 2016). The first cluster involves the cognitive restructuring of the behavior, whereby harmful actions are reframed to make them appear morally acceptable. This includes: (1) *moral justification*, which refers to harmful acts being portrayed as serving socially or morally valuable purposes (e.g., “People deserve to know what she’s really like”); (2) *euphemistic labeling*, which refers to the use of minimizing language to obscure the true nature of the act (e.g., “It was just a joke”); and (3) *advantageous comparison*, where the behavior is compared to more extreme actions to make it look less severe (e.g., “At least he didn’t force her to do anything”). The second cluster refers to the obscuring of personal agency, through which individuals minimize their own responsibility in harmful acts. This includes: (4) *displacement of responsibility*, which involves attributing responsibility to authority figures or external pressures (e.g., “I only did it because everyone was pressuring me”); and (5) *diffusion of responsibility*, where responsibility is diluted across a group or collective action, diminishing individual accountability (e.g., “It wasn’t just me, everyone was sharing it”). The third cluster involves disregarding or distorting consequences, that is, reducing awareness of, or concern for, the harm caused. This includes: (6) *disregarding or minimizing consequences*, where the impact on the victim is ignored, minimized or questioned (e.g., “She’ll get over it”). The fourth cluster concerns the devaluation of the victim, which consists of dehumanizing or blaming the victim in ways that reduce empathy. This includes: (7) *dehumanization*, the process of stripping victims of their human qualities and treating them as objects (e.g., “She’s just something for people to look at”); and (8) *attribution of blame*, the act of holding the victim responsible for provoking or deserving the harmful behavior directed at them (victim blaming) (e.g., “She shouldn’t have sent those pictures in the first place”).

According to socio-cognitive theory, individuals’ actions and moral judgments are shaped by their interactions, experiences, and socialization processes within specific social contexts (Bandura, 2016). This means that each of these mechanisms may operate differently, depending on both the characteristics of the abusive situation (e.g., sextortion vs. NCIIS) and those of the person evaluating it (e.g., sociodemographic variables). In this regard, existing evidence suggests that moral disengagement mechanisms are activated in IBSA situations (Pina et al., 2021) and that their activation may be shaped by variables such as gender (Ojeda et al., 2024) and age (Chen & Sutton, 2025). However, although such differences have been identified at a broad level, it remains unclear whether they reflect a general variation in overall moral disengagement or distinct patterns in the activation of specific mechanisms across age and gender groups. Clarifying these differences is important, as it may help explain how IBSA becomes cognitively normalized and socially tolerated across different groups, while also informing more targeted prevention and intervention efforts aimed at challenging the specific cognitive processes that sustain this form of abuse. The following section explores how gender and age may shape the activation of these mechanisms.

### 1.2. The Role of Gender and Age in Moral Disengagement Mechanisms in IBSA

Gender is a particularly important factor in understanding how moral disengagement may operate within the IBSA context. Specifically, existing evidence shows that men generally exhibit higher levels of moral disengagement than women (Martínez-Bacaicoa et al., 2024a; Piccardi et al., 2023). This is not surprising, as women tend to experience the consequences of IBSA more severely, which may, in turn, foster higher levels of empathy and a lower tolerance of this form of violence (Adair & Senn, 2026; Walker & Sleath, 2017). However, the influence of gender on moral disengagement in IBSA contexts does not appear to be limited to the extent to which it is activated, but rather to how it is activated. In this line, Ojeda et al. (2024) analyzed the activation of the different moral disengagement mechanisms in response to NIIC and found differentiated patterns depending on the gender of the person appearing in the images. In particular, when the person depicted in the images was a boy, mechanisms related to the minimization or distortion of consequences became more salient (e.g., “It won’t really harm him”, “He’ll get over it”). In contrast, when the victim was a girl, certain forms of cognitive restructuring—such as euphemistic labeling or moral justification—became more prominent (e.g., “It was just a picture”). Furthermore, research has shown that the dissemination of sexual images depicting girls or women often elicits victim-blaming responses (Pacilli et al., 2024). This tendency is theoretically plausible if we consider that, in many sociocultural contexts, male sexuality tends to be less socially sanctioned and more permissively interpreted; so, the NCII of boys may be more easily perceived as something “less serious” or as having lower reputational and social impact (Amudhan et al., 2024; Naezer & van Oosterhout, 2021). In the case of women, precisely because they are socially expected to be blamed for the existence of such images (Mandau, 2020; Ringrose et al., 2022b), the activation of moral disengagement mechanisms based on the attribution of responsibility to the victim may be facilitated. These findings therefore seem to reflect the influence of gender socialization and sexual double standards in the way digital sexual violence is interpreted and moralized. Therefore, it is especially relevant to broaden the focus of analysis and explore not only whether these mechanisms are differentially activated depending on who appears in the image but also depending on the gender of the observer. Likewise, it is also important to explore this in other forms of IBSA that are equally concerning but less well-known and less well-understood, such as sextortion.

As well as gender, age is another key sociodemographic factor to consider in relation to moral disengagement, as the ways in which individuals interpret harm, assign responsibility, and judge the acceptability of certain behaviors are progressively shaped through socialization and experience across the lifespan (Luo & Bussey, 2023; Nucci et al., 2018). Existing research suggests that younger individuals tend to report higher levels of moral disengagement (Chen & Sutton, 2025); however, this association has not yet been directly examined in the context of IBSA. Even so, the available evidence appears consistent with this possibility, as younger individuals seem more likely to minimize the harm caused by IBSA (Umbach & Henry, 2025), attribute greater responsibility to victims (Sciacca et al., 2023), and express more permissive attitudes towards this form of abuse (Döring, 2014; Livingstone & Görzig, 2014). As in the case of gender, this does not necessarily imply that all moral disengagement mechanisms operate uniformly across age groups; rather, some may be more salient at earlier stages of life, whereas others may become more relevant later on. Adolescence and emerging adulthood, for instance, are often linked to greater risk-taking and stronger peer influence (Castellanos et al., 2023; Ciranka & Van Den Bos, 2021). These factors may facilitate mechanisms that minimize or downplay the consequences of harmful behavior, such as framing it as “not a big deal” or assuming that “everyone does it”. In contrast, middle and late adulthood are associated with greater sensitivity to the emotional and relational consequences of one’s own actions, as reflected in slight increases in emotional empathy and emotional competence (Pollerhoff et al., 2022). As a result, adults may be less inclined to minimize harm, but more likely to rely on cognitive restructuring mechanisms that reinterpret the behavior in ways that make it seem socially acceptable. Although these developmental differences remain hypothetical, examining them could help clarify not only whether moral disengagement varies across age, but also how specific mechanisms may be activated differently at different stages of the lifespan. This might be particularly relevant in the context of IBSA, as most existing research has focused on adolescents and young people (e.g., Gámez-Guadix et al., 2022, 2023; Ojeda et al., 2024), while far less is known about how these dynamics operate in older age groups. Available evidence indicates that IBSA also occurs among adults, though less frequently (Miguel-Alvaro et al., 2025), highlighting the need to examine how the mechanisms that facilitate its perpetration operate at different stages of life.

Taken together, this evidence suggests that both gender and age play an important role in shaping moral disengagement in IBSA contexts. Research indicates that gender biases and stereotypes emerge early in development and continue to evolve across the lifespan (Geraci et al., 2025). At the same time, the cognitive, social, and moral processes through which individuals interpret harm and responsibility also undergo important developmental changes (Nucci et al., 2018). Consequently, the influence of gender on moral disengagement may not be uniform across developmental stages, but may vary as gender-related beliefs interact with broader changes in moral reasoning and social judgment. In this regard, gender differences in the activation of moral disengagement mechanisms may, for example, become less pronounced as individuals age, as their reasoning becomes less influenced by social norms (Castellanos et al., 2023). These influences may be reflected not only in overall levels of moral disengagement, but also in the specific mechanisms through which abusive behaviors are cognitively justified, minimized, or normalized. However, despite growing evidence regarding the separate roles of gender and age, little is known about how these variables jointly contribute to different patterns of moral disengagement mechanism activation in IBSA contexts. Examining their combined influence may therefore help clarify not only whether moral disengagement varies according to age and gender, but also whether specific mechanisms are differentially activated across demographic groups.

### 1.3. The Present Study

The aim of the present study is to examine how the activation of different mechanisms of moral disengagement in response to IBSA varies according to gender and age across the lifespan. More specifically, this study focused on scenarios depicting sextortion and NCIIS involving male-perpetrated abuse against women, reflecting the gendered nature of this form of violence. Accordingly, this study addressed three objectives: (1) to identify the activation of distinct moral disengagement mechanisms in NCIIS and sextortion scenarios; (2) to examine how these mechanisms vary as a function of gender and age; and (3) to analyze the interaction between these variables in shaping moral disengagement in such contexts. Based on the existing literature, the following hypotheses were proposed.

**H1.**  *For both NCIIS and sextortion scenarios, attribution of blame and euphemistic labeling are expected to be among the most strongly activated moral disengagement mechanisms.*

**H2.**  *For both NCIIS and sextortion scenarios, men and younger individuals will report higher levels of moral disengagement than women and older individuals, respectively.*

**H3.**  *For both NCIIS and sextortion scenarios, gender differences in the activation of moral disengagement mechanisms will be more pronounced among younger individuals and will progressively decrease across the lifespan.*

A vignette-based methodology was employed to standardize the presentation of IBSA scenarios and to reduce social desirability bias in participants’ responses (Hainmueller et al., 2015). By examining these patterns, this study aims to contribute to a more nuanced understanding of how IBSA may be cognitively justified, minimized, or normalized across different sociodemographic groups. In doing so, it may also help inform more targeted prevention and intervention strategies aimed at challenging the specific cognitive processes that sustain this form of abuse.

## 2. Methods

### 2.1. Sample

The sample consisted of 2343 participants (68.2% women, 29.5% men, 1.8% nonbinary, and 0.4% who preferred not to disclose their gender). Participants ranged in age from 14 to 74 years (*M_age_* = 25.86, SD = 9.96, *M_o_* = 19). Regarding sexual orientation, 69.4% (*n* = 1633) identified as heterosexual, 20.0% (*n* = 468) as bisexual, 5.3% (*n* = 124) as lesbian or gay, and 2.0% as another sexual orientation not listed in the questionnaire. The remaining 3.0% (*n* = 71) preferred not to disclose their sexual orientation. Regarding their country of origin, 89.7% reported being born in Spain and 10.3% in another country. When asked about the time they spend using technology daily during the week, 14.7% reported spending one hour or less per day, 33.5% approximately two hours, 25.5% three hours, and 26.2% four or more hours.

### 2.2. Procedure

Data collection took place between May 2021 and January 2022. Participants were invited to take part in an online study through two main channels: (1) email invitations sent to Spanish educational institutions and universities, and (2) dissemination via social media platforms, primarily Facebook and Instagram. Individuals who expressed interest were directed to an online information page that outlined the purpose of this study and provided contact details for the research team. Access to the questionnaire was provided via a link to the Qualtrics platform, where participants completed the survey, which took approximately 25–30 min. Prior to participation, respondents were required to provide informed consent after being informed of the voluntary nature of this study, the confidentiality of their responses, and their right to withdraw at any time or omit any question without penalty. Only those who consented were able to proceed to the questionnaire. This study formed part of a broader research project on Technology-Facilitated Sexual Violence (TFSV) approved by the Ethics Committee of the Autonomous University of Madrid.

### 2.3. Measures

Moral disengagement: Moral disengagement was assessed using a vignette-based measure. Participants were presented with two scenarios depicting distinct forms of IBSA, namely sextortion and NCIIS. In all scenarios, the perpetrator was described as a man and the victim as a woman. To control for extraneous variables known to influence perceptions of gender-based violence, the vignettes were designed to exclude information about the victim’s emotional responses, behavioral reactions, and physical appearance. The content of each vignette is described in the Supplementary Materials. Following each vignette, participants responded to eight items rated on a 5-point Likert scale ranging from 0 (strongly disagree) to 4 (strongly agree). Each item corresponded to one of the eight mechanisms of moral disengagement: moral justification, euphemistic labeling, advantageous comparison, displacement of responsibility, diffusion of responsibility, distortion of consequences, and dehumanization of the victim. Previous research conducted with Spanish samples (Martínez-Bacaicoa et al., 2024a) has shown an overall good model fit across types of violence, with CFI values above 0.90 and standardized Root mean Square Residuals (SRMR) below 0.08. The measure also showed adequate internal consistency, with Cronbach’s alpha coefficients ranging from 0.82 to 0.93. In the present study, internal consistency was satisfactory for both sextortion (α = 0.88) and NCIIS (α = 0.83).

### 2.4. Statistical Analysis

Descriptive statistics were computed for all study variables, along with Spearman’s rank-order correlations to examine their associations. Given the very small proportion of nonbinary participants (1.8%) and individuals who did not disclose their gender (0.4%), these cases were excluded from subsequent analyses to ensure sufficient statistical power and the stability of group comparisons. Gender differences in moral disengagement mechanisms were initially examined using the Mann–Whitney U test. To further examine the effects of gender on moral disengagement while controlling for age, a multivariate general linear model (MANCOVA) was conducted. This approach was preferred to reduce the inflation of Type I error associated with multiple univariate analyses. Gender was included as a fixed factor, age (mean-centered) as a covariate, and the interaction between age and gender was included to assess potential moderation effects. The Box’s M test was statistically significant (*M* = 1744.61; *F* = 48.11; *p* < 0.001), indicating a violation of the assumption of equality of variance–covariance matrices across groups. Similarly, Levene’s test results were significant for all dependent variables (*p*s < 0.001), suggesting that the assumption of homoscedasticity was not met. Given these violations, several methodological decisions were implemented to ensure the robustness and validity of the findings. Firstly, Pillai’s Trace was used to evaluate multivariate effects instead of Wilks’ Lambda, as it is considered the most robust statistic under violations of homogeneity of covariance matrices and unequal group sizes. Secondly, a more conservative significance threshold was adopted to reduce the risk of Type I error. Specifically, effects were considered statistically significant at *p* < 0.01 instead of the conventional 0.05 level. No additional multiple-comparison corrections were applied, as the analyses were conducted within a single multivariate framework.

Approximately 5% of the data were missing. The pattern of missingness was evaluated using Little’s Missing Completely at Random (MCAR) test, which was non-significant, χ^2^(75) = 68.38, *p* = 0.69, indicating that the data were missing completely at random. Therefore, listwise deletion was applied to handle missing values (Little, 1988). Although alternative modern approaches, such as multiple imputation, were considered, listwise deletion was preferred given the very low rate of missingness (<5%), under which this method provides unbiased and efficient estimates without adding unnecessary model complexity (Allison, 2001; Graham, 2009). All analyses were conducted using IBM SPSS Statistics version 28.

## 3. Results

Spearman’s correlations (Table 1) revealed that all moral disengagement mechanisms in NCIIS and sextortion were positively and significantly interrelated (r_s_ = 0.17 to 0.62, *p* < 0.001). The strongest associations emerged within the same IBSA domain, particularly between diffusion of responsibility and advantageous comparison in sextortion. Cross-domain correlations were also moderate to strong, indicating consistency of moral disengagement across behaviors. Age showed small but significant negative correlations with all variables (r_s_ = −0.09 to −0.18, *p* < 0.01–0.001), suggesting that younger participants reported higher levels of moral disengagement.

Mann–Whitney U (Table 2) tests indicated that men scored significantly higher than women across all variables of moral disengagement in both NCIIS and sextortion (all *p* < 0.001). The largest differences were observed in euphemistic labeling for NCIIS and attribution of blame for NCIIS and sextortion.

### Differential Effects of Gender and Age on IBSA

Multivariate analyses of covariance were conducted to examine the effects of gender and age on both NCIIS and sextortion-related behaviors. Using Pillai’s Trace, results showed that gender had a significant multivariate effect in both models with men showing higher scores across domains. In terms of magnitude, the effect was moderate for NCIIS behaviors (*V* = 0.085, *F*(8, 1489) = 17.30, *p* < 0.001; *n_p_*^2^ = 0.085) and smaller for sextortion (*V* = 0.043, *F*(8, 1552) = 8.63, *p* < 0.001; *n_p_*^2^ = 0.043), suggesting that gender accounted for more variance in moral justification scores of NCIIS than for sextortion. Similarly, age was significantly associated with both outcomes, with moral justification decreasing among older participants. These effects were slightly larger for NCIIS behaviors (*V* = 0.026, *F*(8, 1489) = 4.99, *p* < 0.001; *n_p_*^2^ = 0.026) than sextortion (*V* = 0.020, *F*(8, 1552) = 4.02, *p* < 0.001; *n_p_*^2^ = 0.020), suggesting that while age is a reliable predictor, its impact on the variance in these behaviors is limited and likely conditioned by other complex interpersonal differences. Notably, the interaction between gender and age was significant for NCIIS (*V* = 0.016, *F*(8, 1489) = 2.98, *p* = 0.003; *n_p_*^2^ = 0.016), indicating that age-related differences varied by gender in this domain, whereas this interaction only reached marginal significance for sextortion under the conservative threshold (*p* < 0.01), (*V* = 0.012, *F*(8, 1552) = 2.26, *p* = 0.021; *n_p_*^2^ = 0.012). This pattern further supports the notion that, while demographic factors anchor baseline differences, the expression of these behaviors—especially in sextortion—is shaped by broader interpersonal and contextual factors that extend beyond simple age categorization.

Follow-up univariate analyses and parameter estimates were examined to clarify the univariate effects (Table 3). With regard to gender, results revealed a consistent and significant effect across all outcomes in both models. Specifically, men reported higher levels of all measured mechanisms compared to women in both NCIIS and sextortion contexts (all *p*s < 0.001). Age also emerged as a significant predictor across most outcomes, with older participants reporting lower levels of moral disengagement mechanisms. An exception was observed for disregarding or minimizing consequences in the sextortion model, which did not show a meaningful decrease with age (*p* = 0.014), suggesting that this specific mechanism remains relatively stable across age in comparison with the others.

**Figure 1:**
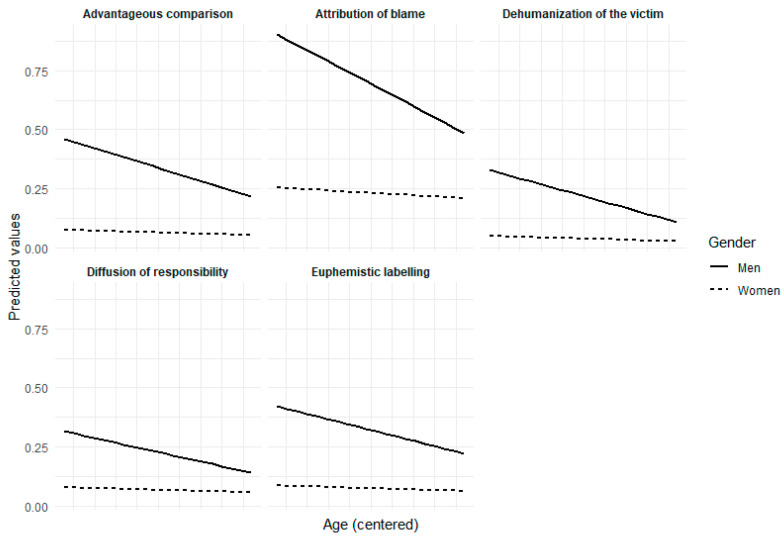
Significant Interaction Terms for NCIIS.

**Figure 2:**
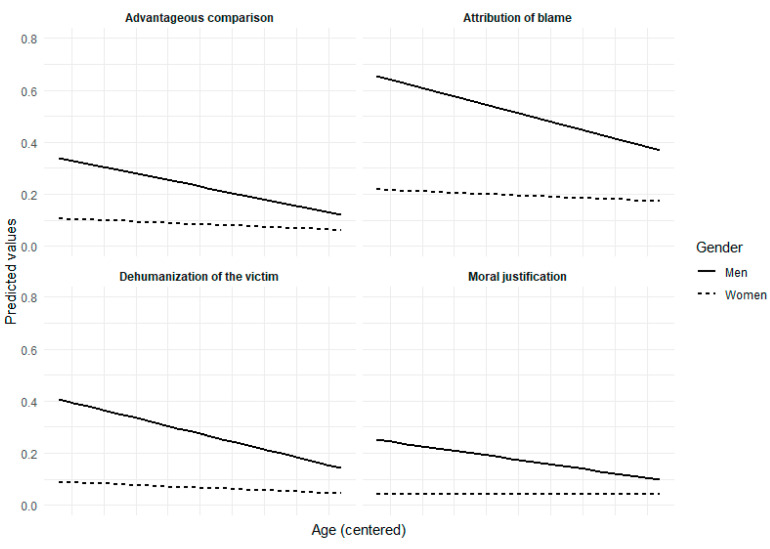
Significant Interaction Terms for Sextortion.

With respect to the interaction between gender and age, several significant effects emerged, indicating that age-related decreases in moral disengagement mechanisms differed by gender across multiple domains. As shown in Table 3, most interaction terms were positive, suggesting that although moral disengagement tended to decline with age for both men and women, this decrease was generally steeper among men. In other words, gender differences in these mechanisms were more pronounced at younger ages and became attenuated over time. This trend was non-significant for displacement of responsibility and for disregarding or minimizing consequences, both in NCIIS and in sextortion. This pattern suggests that the decline occurred at a similar rate for both men and women, while men maintained significantly higher levels across the lifespan. A similar pattern was also observed for moral justification in NCIIS as well as for euphemistic labeling and diffusion of responsibility in sextortion. Figures illustrating the significant interaction terms for NCIIS and sextortion are presented in Figure 1 and Figure 2, respectively.

## 4. Discussion

IBSA is receiving increasing scholarly attention. Research has highlighted the role of moral disengagement mechanisms in the justification of this form of violence and has begun to explore gender differences in these processes (e.g., Ojeda et al., 2024). However, considerably less is known about how these mechanisms vary across the lifespan, particularly when considering the combined influence of age and gender. The present study aimed to address this gap by examining how gender and age shape the selective activation of different moral disengagement mechanisms in response to hypothetical NCIIS and sextortion scenarios. The results are broadly consistent with our hypotheses and indicate that victim-blaming and euphemistic labeling are among the most strongly endorsed moral disengagement mechanisms (H1), that younger men exhibit the highest levels of moral disengagement (H2), and that gender differences are more pronounced among younger participants and tend to decrease across the lifespan (H3).

The fact that the findings consistently show that men report higher levels of moral disengagement than women across both forms of IBSA aligns with prior literature suggesting that men are more likely to justify these forms of violence (e.g., Ojeda et al., 2024; Pina et al., 2021). However, although both NCIIS and sextortion are shaped by gender dynamics, the magnitude of these differences appears to vary across these contexts. The results indicate that gender differences are more pronounced in cases of NCIIS, suggesting that this behavior may be particularly normalized among men. In this regard, NCIIS may be perceived as less confrontational and more embedded in everyday digital practices, such as sharing or forwarding sexualized content, which may be especially normalized in male-dominated online spaces (e.g., manosphere, private group chats on Telegram, video streaming websites, online community forums) (Franco et al., 2024; Henry et al., 2026; Semenzin & Bainotti, 2020). In this sense, NCIIS has been described as part of “economies” of male prestige, in which images of girls are displayed, exchanged, or collected as “trophies”, thereby reinforcing this form of violence (Ringrose et al., 2022b). Within these environments, men may not necessarily act as primary perpetrators but are nonetheless exposed to these practices either passively (e.g., when they receive such content unsolicited from peers) (Minihan et al., 2025) or through active engagement (e.g., by visiting online communities to view non-consensual shared content) (Henry et al., 2026). This exposure may not only reinforce certain gender norms but may also contribute to processes of moral disengagement through which men might come to justify these forms of violence (Esposito et al., 2022). In contrast, sextortion tends to be less visible within everyday male digital interactions, which may limit its normalization through observation. Moreover, it typically involves more explicit coercion and a clearer threat of harm, making it more difficult to trivialize. Additionally, although gendered dynamics have been documented, sextortion can occur through indiscriminate, large-scale strategies, such as mass phishing emails, where victims are not specifically targeted and frequently include men (Ray & Henry, 2025). This broader exposure may increase men’s likelihood of having experienced the victim role, which could, in turn, help explain reduced gender differences in the justification of such behaviors compared to NCIIS.

Beyond these overall differences, a closer examination of specific moral disengagement mechanisms reveals more gendered patterns. Across both contexts, victim blaming (e.g., “She shouldn’t have sent those pictures in the first place”) emerges as the mechanism which shows the largest gender differences, with men endorsing it more frequently than women. This finding aligns with a substantial body of IBSA research in which victim blaming has been consistently documented, underscoring its persistence as a common justificatory logic among men (e.g., Flynn et al., 2023). This logic is underpinned by beliefs rooted in sexual double standards and rape myth acceptance, which position women as responsible for preventing victimization, thereby shifting accountability away from perpetrators (Adair & Senn, 2026). The fact that these gender differences emerge consistently across both IBSA contexts suggests that victim blaming operates as a relatively transversal mechanism among men, one that may be less sensitive to the specific features of each form of abuse and instead functions as a more generalized justificatory “default” in cases of sexual violence against women. As such, it may be understood as a foundational element in the male justification of sexual violence against women, onto which other mechanisms are layered depending on the characteristics of the situation. In addition, in the case of NCIIS, significant gender differences were found in euphemistic labeling (e.g., “It was just a joke”), with men relying on this mechanism more frequently than women. This finding is consistent with the results reported by Ojeda et al. (2024), who observed that when the shared image depicted a girl, as in the present study, mechanisms based on cognitive restructuring, such as euphemistic labeling, were particularly activated. This mechanism operates by redefining the act itself, thereby making it more morally acceptable. Accordingly, existing evidence shows that, in groups of boys and men, practices such as forwarding or displaying intimate images are often framed as a “joke” or as “not that serious” (Ringrose et al., 2022a; Walker et al., 2021). These findings suggest that the normalization of NCIIS is sustained through such discursive processes, which may form part of a broader collective narrative that increases the acceptability of these behaviors.

With regard to age, younger participants showed higher levels of moral disengagement. This finding is consistent with previous research indicating that younger individuals tend to show higher levels of moral disengagement (Chen & Sutton, 2025) and this may be interpreted from a developmental perspective. Adolescence is characterized by heightened susceptibility to peer influence, less mature moral reasoning (Castellanos et al., 2023), and the exploration of sexual norms (Hegde et al., 2022). As individuals mature, moral reasoning typically becomes more advanced, empathy deepens, and perspective-taking abilities improve (Romera et al., 2021), which may reduce the activation of moral disengagement mechanisms. However, a closer examination of specific moral disengagement mechanisms and the interaction between gender and age reveals patterns that cannot be fully explained by age-related differences alone.

Firstly, the mechanism-specific analysis shows that age-related differences are neither consistent nor statistically significant across all forms of moral disengagement. Specifically, the tendency to degrade or minimize the consequences (e.g., “She’ll get over it”) in sextortion remains relatively stable across age groups. This suggests that, even when the overall tendency to justify such behaviors lower among older individuals, certain mechanisms appear to follow a more stable trajectory across age groups. As discussed earlier, one possible explanation is that IBSA against women is perceived, even at younger ages, as a serious and harmful behavior (Mandau, 2020; Ringrose et al., 2022b), leaving less room for denying its impact. However, additional research, particularly longitudinal studies on moral disengagement in sextortion is needed to determine whether these patterns reflect developmental processes, cohort effects, or generational differences in digital socialization and to better contextualize these findings.

Secondly, the analysis of the interaction between gender and age indicated that the decline in moral disengagement across age groups was shaped by gender, particularly in NCIIS contexts. More specifically, moral disengagement tends to decrease with age among men, while it remains relatively stable among women. This pattern may be understood in light of gendered socialization processes: as previously stated, young men often socialize in peer groups where the IBSA—and particularly the non-consensual sharing of sexualized pictures—is relatively common. These environments may sustain higher levels of moral disengagement in early stages, with comparatively lower levels observed among older individuals, potentially reflecting differences in exposure to such peer dynamics. As a result, women may show a less pronounced pattern across age groups in moral disengagement in relation to IBSA, given that their baseline levels are already lower (Ojeda et al., 2024). However, the influence of gender is not consistent across all mechanisms. In particular, mechanisms such as the minimization of consequences and the displacement of responsibility do not appear to show gender-based differences in their association with age, in either sextortion or NCIIS contexts. Building on the previously noted stability of consequence minimization across age groups, the present findings further indicate that this pattern is similar for both men and women, although men tend to endorse this mechanism more frequently overall. This suggests that this is a justificatory mechanism that, while gendered in its intensity, does not appear to be closely tied to processes that evolve over time (e.g., peer online groups in which sexualized images are shared or the use of humor to frame these behaviors). Rather, these practices seem to be justified through other mechanisms, such as euphemistic labeling or victim blaming, which are more directly embedded in these social contexts.

### 4.1. Limitations and Future Directions

This study presents several limitations that should be considered when interpreting the findings. Firstly, the use of self-report measures may have introduced social desirability bias, despite participants being assured of the anonymity and confidentiality of their responses. Given the sensitive nature of the topic, participants may have underreported socially undesirable attitudes or responses, affecting the accuracy of the findings. Secondly, given the exploratory nature of this study, some potentially relevant variables were not included. Factors such as prior experiences with IBSA (e.g., as victims, bystanders, or perpetrators), individual ideological orientations (e.g., sexist attitudes) and different victim–perpetrator gender configurations (e.g., same-gender victim–perpetrator dyads) may play an important role in shaping moral disengagement and should be examined in future research. Thirdly, although this study focused on two prominent forms of IBSA, it did not address other emerging manifestations that are rapidly gaining relevance. In particular, AI-generated sexualized images (e.g., deepfakes) represent a growing concern and introduce new challenges, as the material involved may not correspond to real events. Fourthly, this study is cross-sectional and does not assess the relationship between moral disengagement and the actual perpetration of, or engagement in, IBSA behaviors. Although age differences were observed, the cross-sectional design does not allow us to disentangle developmental changes from cohort effects or generational differences in digital socialization. Longitudinal research is needed to examine the temporal relationship between moral disengagement and experiences of IBSA, both as a perpetrator and as a bystander. Fifthly, the sample was recruited primarily through social media platforms and educational institutions, which may have introduced self-selection bias. Individuals with a particular interest in the topic or greater engagement with online environments may have been more likely to participate, potentially limiting the representativeness of the sample and the generalizability of the findings. In addition, the sample consisted predominantly of Spanish participants. As attitudes toward sexuality, gender norms, consent, and digital behaviors are shaped by sociocultural contexts, the findings may reflect characteristics specific to the Spanish cultural setting and may not be fully generalizable to other countries or cultural groups. Cross-cultural research is needed to examine the extent to which these findings are replicated across different sociocultural contexts. Finally, women were substantially overrepresented in the sample, which may have influenced the overall results and limited the extent to which the findings reflect the perspectives of men and other gender groups. Consequently, caution is warranted when interpreting gender-related patterns. Furthermore, although nonbinary participants were initially recruited, they were excluded from the gender-stratified analyses due to the limited sample size, which did not allow for statistically robust comparisons. This decision reduces the inclusiveness and generalizability of the findings across the full spectrum of gender identities. Future research should aim to recruit larger and more diverse samples to enable the inclusion of nonbinary individuals in the analyses and to better understand potential differences and similarities across gender groups.

### 4.2. Practical Implications

These findings carry important implications for prevention and intervention strategies. Firstly, they suggest that prevention could usefully focus particularly on young men, who exhibit higher levels of moral disengagement and are therefore more likely to justify, engage in, or tolerate these forms of violence. Interventions targeting this group may benefit from placing particular emphasis on challenging core beliefs that appear to be central to male justifications of IBSA, particularly victim blaming, which emerges consistently across both sextortion and NCIIS. In the case of NCIIS, prevention efforts could also explicitly address the tendency to frame these behaviors as “jokes” or as harmless, given that euphemistic labeling appears to be especially prevalent among young men. While other justificatory mechanisms should not be overlooked, the present findings suggest that these beliefs may be particularly salient within specific groups and therefore warrant targeted attention. By contrast, mechanisms such as the minimization of consequences appear to be less sensitive to age and gendered social dynamics, possibly because the harmful nature of these behaviors is more broadly recognized. As such, prevention strategies may benefit from combining targeted approaches that address group-specific justificatory processes with broader efforts aimed at reinforcing awareness of harm and responsibility across populations. These targeted approaches may be complemented by broader educational, regulatory, and victim-support initiatives aimed at preventing IBSA and mitigating its consequences across different social and institutional contexts.

## 5. Conclusions

This study examines IBSA by analyzing how age and gender jointly shape the moral disengagement mechanisms that justify its occurrence across age groups. The findings particularly underscore that young men exhibit the highest levels of moral disengagement in relation to both NCIIS and sextortion, reinforcing the idea that IBSA is a strongly gendered form of violence. These patterns may be associated with the increasing prevalence of online dynamics, such as the normalization of sharing intimate images in group chats or the use of humor to frame these behaviors, which can reinforce underlying beliefs linked to moral disengagement mechanisms such as victim blaming and euphemistic labeling (Durán-Guerrero & Sánchez-Jiménez, 2025; Esposito et al., 2022). Considering these factors across different stages of life can inform the design of prevention and intervention strategies that are sensitive to both age and gender while directly targeting the cognitive processes that sustain these forms of violence. Ultimately, addressing these justificatory processes is important for challenging the social acceptance of IBSA and reducing its prevalence.

## Figures and Tables

**Table 1:** Spearman’s Correlations Among All Study Variables.

Variable	1	2	3	4	5	6	7	8	9	10	11	12	13	14	15	16
1. NCIIS_ lab	—															
2. NCIIS_ just	0.58 ***	—														
3. NCIIS_ comp	0.50 ***	0.49 ***	—													
4. NCIIS_disp	0.36 ***	0.43 ***	0.35 ***	—												
5. NCIIS_ diff	0.48 ***	0.50 ***	0.50 ***	0.49 ***	—											
6. NCIIS_ cons	0.46 ***	0.50 ***	0.47 ***	0.35 ***	0.46 ***	—										
7. NCIIS_ blame	0.33 ***	0.29 ***	0.34 ***	0.22 ***	0.30 ***	0.28 ***	—									
8. NCIIS_ deh	0.41 ***	0.50 ***	0.48 ***	0.36 ***	0.48 ***	0.51 ***	0.42 ***	—								
9. SEX_ lab	0.36 ***	0.43 ***	0.40 ***	0.32 ***	0.38 ***	0.36 ***	0.21 ***	0.36 ***	—							
10. SEX_ just	0.40 ***	0.53 ***	0.49 ***	0.44 ***	0.49 ***	0.45 ***	0.26 ***	0.51 ***	0.60 ***	—						
11. SEX_ comp	0.38 ***	0.47 ***	0.49 ***	0.41 ***	0.54 ***	0.44 ***	0.28 ***	0.54 ***	0.53 ***	0.57 ***	—					
12. SEX_disp	0.31 ***	0.33 ***	0.27 ***	0.62 ***	0.41 ***	0.35 ***	0.17 ***	0.37 ***	0.40 ***	0.44 ***	0.45 ***	—				
13. SEX_ diff	0.41 ***	0.42 ***	0.41 ***	0.45 ***	0.56 ***	0.35 ***	0.25 ***	0.43 ***	0.50 ***	0.55 ***	0.61 ***	0.55 ***	—			
14. SEX_ cons	0.36 ***	0.38 ***	0.36 ***	0.36 ***	0.42 ***	0.54 ***	0.26 ***	0.48 ***	0.47 ***	0.56 ***	0.55 ***	0.46 ***	0.51 ***	—		
15. SEX_ blame	0.34 ***	0.36 ***	0.39 ***	0.28 ***	0.33 ***	0.30 ***	0.50 ***	0.37 ***	0.41 ***	0.43 ***	0.42 ***	0.34 ***	0.42 ***	0.35 ***	—	
16.SEX_ deh	0.33 ***	0.40 ***	0.44 ***	0.29 ***	0.39 ***	0.44 ***	0.31 ***	0.56 ***	0.49 ***	0.57 ***	0.49 ***	0.41 ***	0.52 ***	0.51 ***	0.56 ***	—
17. Age	−0.12 ***	−0.09 ***	−0.14 ***	−0.09 **	−0.09 **	−0.11 ***	−0.14 ***	−0.12 ***	−14 ***	−0.15 ***	−0.16 ***	−0.14 ***	−0.16 ***	−0.11 ***	−0.17 ***	−18 ***
*Mean*	0.14	0.08	0.14	0.20	0.12	0.13	0.37	0.09	0.11	0.09	0.13	0.19	0.13	0.12	0.29	0.13
SD	0.55	0.40	0.56	0.67	0.48	0.54	0.91	0.44	0.51	0.44	0.54	0.62	0.52	0.58	0.78	0.56

Note: NCIIS = Non-Consensual Intimate Image Sharing, SEX = Sextortion, lab = Euphemistic labeling, just = moral justification, comp = advantageous comparison, disp = displacement of responsibility, diff = diffusion of responsibility, cons = disregarding or minimizing of consequences, blame = attribution of blame, deh = dehumanization of the victim. *** *p* < 0.001; ** *p* < 0.01.

**Table 2:** Gender Differences Across Study Variables Based on Mann–Whitney U Test.

	Women	Men		
	*Mean* (SD)	*Mean* (SD)	*U*	*Z*
1. NCIIS_lab	0.07 (0.39)	0.31 (0.79)	205,599	−8.37 ***
2. NCIIS_just	0.04 (0.29)	0.18 (0.58)	214,067	−7.19 ***
3. NCIIS_comp	0.06 (0.37)	0.33 (0.84)	204,636	−8.81 ***
4. NCIIS_disp	0.14 (0.54)	0.34 (0.83)	213,131	−5.36 ***
5. NCIIS_diff	0.07 (0.37)	0.22 (0.64)	215,179	−6.33 ***
6. NCIIS_cons	0.08 (0.45)	0.24 (0.69)	215,258	−6.09 ***
7. NCIIS_blame	0.23 (0.70)	0.67 (1.21)	193,951	−8.23 ***
8. NCIIS_deh	0.04 (0.27)	0.21 (0.687)	215,757	−7.00 ***
9. SEX_lab	0.08 (0.44)	0.19 (0.65)	246,427	−4.75 ***
10. SEX_just	0.04 (0.29)	0.17 (0.62)	243,738	−5.99 ***
11. SEX_comp	0.08 (0.43)	0.23 (0.71)	243,188	−5.29 ***
12. SEX_disp	0.15 (0.55)	0.28 (0.76)	246,475	−3.53 ***
13. SEX_diff	0.09 (0.41)	0.23 (0.71)	246,738	−4.09 ***
14. SEX_cons	0.09 (0.50)	0.20 (0.73)	248,227	−4.32 ***
15. SEX_blame	0.19 (0.62)	0.51 (1.02)	225,804	−6.96 ***
16. SEX_deh	0.09 (0.39)	0.27 (0.79)	237,424	−7.00 ***

Note: NCIIS = Non-Consensual Intimate Image Sharing, SEX = Sextortion, lab = Euphemistic labeling, just = moral justification, comp = advantageous comparison, disp = displacement of responsibility, diff = diffusion of responsibility, cons = disregarding or minimizing of consequences, blame = attribution of blame, deh = dehumanization of the victim. *** *p* < 0.001.

**Table 3:** Parameter estimates for the Non-Consensual Intimate Image Sharing and Sextortion.

	Non-Consensual Intimate Image Sharing					Sextortion				
	B	SE	*t*	*p*	95% CI	B	SE	*t*	*p*	95% CI
Lab										
Gender	−0.247	0.031	−8.08	<0.001	[−0.307, −0.187]	−0.114	0.028	−4.06	<0.001	[−0.170, −0.059]
Age	−0.009	0.002	−3.94	<0.001	[−0.014, −0.005]	−0.005	0.002	−2.59	0.010	[−0.009, −0.001]
G × A	0.008	0.003	2.76	0.006	[0.002, 0.014]	0.005	0.003	1.93	0.053	[0.000, 0.010]
Just										
Gender	−0.146	0.023	−6.47	<0.001	[−0.190, −0.102]	−0.132	0.023	−5.77	<0.001	[−0.177, −0.087]
Age	−0.005	0.002	−2.67	0.008	[−0.008, −0.001]	−0.007	0.002	−3.99	<0.001	[−0.010, −0.003]
G × A	0.004	0.002	1.99	0.047	[0.000, 0.008]	0.007	0.002	3.26	0.001	[0.003, 0.011]
Comp										
Gender	−0.275	0.031	−8.83	<0.001	[−0.336, −0.213]	−0.146	0.029	−5.01	<0.001	[−0.204, −0.089]
Age	−0.011	0.002	−4.54	<0.001	[−0.015, −0.006]	−0.010	0.002	−4.65	<0.001	[−0.014, −0.006]
G × A	0.010	0.003	3.30	0.001	[0.004, 0.016]	0.008	0.003	3.07	0.002	[0.003, 0.014]
Disp										
Gender	−0.204	0.036	−5.64	<0.001	[−0.275, −0.133]	−0.131	0.034	−3.84	<0.001	[−0.197, −0.064]
Age	−0.008	0.003	−2.81	0.005	[−0.013, −0.002]	−0.009	0.003	−3.70	<0.001	[−0.014, −0.004]
G × A	0.005	0.003	1.34	0.181	[−0.002, 0.011]	0.006	0.003	1.82	0.069	[0.000, 0.012]
Diff										
Gender	−0.161	0.026	−6.10	<0.001	[−0.212, −0.109]	−0.141	0.028	−4.98	<0.001	[−0.196, −0.085]
Age	−0.008	0.002	−4.10	<0.001	[−0.012, −0.004]	−0.008	0.002	−3.95	<0.001	[−0.012, −0.004]
G × A	0.007	0.003	2.66	0.008	[0.002, 0.012]	0.006	0.003	2.36	0.018	[0.001, 0.012]
Cons										
Gender	−0.165	0.030	−5.48	<0.001	[−0.224, −0.106]	−0.116	0.032	−3.63	<0.001	[−0.178, −0.053]
Age	−0.009	0.002	−3.85	<0.001	[−0.013, −0.004]	−0.006	0.002	−2.47	0.014	[−0.010, −0.001]
G × A	0.007	0.003	2.32	0.021	[0.001, 0.012]	0.004	0.003	1.20	0.230	[−0.002, 0.010]
Blame										
Gender	−0.463	0.050	−9.33	<0.001	[−0.560, −0.366]	−0.316	0.042	−7.60	<0.001	[−0.398, −0.235]
Age	−0.019	0.004	−5.02	<0.001	[−0.026, −0.011]	−0.013	0.003	−4.19	<0.001	[−0.019, −0.007]
GxA	0.017	0.005	3.67	<0.001	[0.008, 0.027]	0.011	0.004	2.92	0.004	[0.004, 0.019]
Deh										
Gender	−0.181	0.024	−7.46	<0.001	[−0.229, −0.134]	−0.208	0.030	−6.96	<0.001	[−0.267, −0.149]
Age	−0.010	0.002	−5.59	<0.001	[−0.014, −0.007]	−0.012	0.002	−5.22	<0.001	[−0.016, −0.007]
GxA	0.009	0.002	3.87	<0.001	[0.004, 0.013]	0.010	0.003	3.50	<0.001	[0.004, 0.015]

Note: NCIIS = Non-Consensual Image Sharing, SEX = Sextortion, lab = Euphemistic labeling, just = moral justification, comp = advantageous comparison, disp = displacement of responsibility, diff = diffusion of responsibility, cons = disregarding or minimizing of consequences, blame = attribution of blame, deh = dehumanization of the victim.

## Data Availability

The raw data supporting the conclusions of this article will be made available by the authors on request.

## Supplementary Materials

The following supporting information can be downloaded at: https://www.mdpi.com/article/10.3390/bs16071047/s1, Table S1. Vignettes describing each type of IBSA and moral disengagement items.
