# Supplementary material for: Moral Disengagement Mechanisms in Image-Based Sexual Abuse Against Women: The Role of Age and Gender
Source: Behav Sci (Basel). 2026 Jun 23;16(7):1047. doi: 10.3390/bs16071047 (PMC13405973; doi:10.3390/bs16071047)
Supplement: Supplementary file 1 [file behavsci-16-01047-s001.zip › behavsci-4341505-supplementary.pdf]

Supplementary Material

Table S1. Vignettes describing each type of IBSA and moral disengagement items.

Type of TFVS	Vignette	Moral Disengagement Items
Non-consensual intimate image sharing	George has been dating Mary, a girl with whom he has hit it off very well. He often talks to his friends about how much he likes her, and since he and his friends trust each other, he does not hesitate to tell them about every aspect of the relationship. He tells them that, without even asking, she sends him nude pictures on a regular basis. So, one day, out of curiosity, they ask him to forward them one. Since everyone does this when they go out with someone, he agrees.	1. George is simply entertaining his friend group.<br>2. George's behavior is justified because it was done with the intention of pleasing his friends.<br>3. George's behavior is not serious because he could have done something much worse with the photos.<br>4. People brought up in society like this cannot be blamed for acting like George.<br>5. Many people would have done the same as George, so why shouldn't he?<br>6. George's behavior is harmless.<br>7. Mary is responsible for the fact that a photo of her was shared.<br>8. Mary deserves George's behavior.
Sextortion	A few months ago, Sarah met John through a dating website. After talking for a few weeks, they decided to have a video call to see each other. As they hit it off very well, Sarah did not hesitate to agree to undress in front of him. However, a few days later,	1. John is simply asserting himself.<br>2. John's behavior is justified because it was done with the intention of defending himself<br>3. John's behavior is not serious because he could have done something much worse with the videos.<br>4. People brought up in society like this cannot be blamed for acting like John.

she met someone else, and without any explanation, she stopped talking to John. Angry with Sarah, John decided to send her the following message: “Either you agree to answer me and see me again, or the pictures I took of you during the video call will soon be published on the internet.”	5. Many people would have acted like John, so why wouldn’t he?<br>6. John’s behavior is harmless.<br>7. Sarah is responsible for John’s reaction.<br>8. Sarah deserves John’s reaction.
